# Technical evaluation of different respiratory monitoring systems used for 4D CT acquisition under free breathing

**DOI:** 10.1120/jacmp.v16i2.4917

**Published:** 2015-03-08

**Authors:** Christian Heinz, Michael Reiner, Claus Belka, Franziska Walter, Matthias Söhn

**Affiliations:** ^1^ Department of Radiation Oncology LMU University Hospital D‐81377 Munich Germany

**Keywords:** 4D CT, respiratory monitoring system, free breathing

## Abstract

Respiratory monitoring systems are required to supply CT scanners with information on the patient's breathing during the acquisition of a respiration‐correlated computer tomography (RCCT), also referred to as 4D CT. The information a respiratory monitoring system has to provide to the CT scanner depends on the specific scanner. The purpose of this study is to compare two different respiratory monitoring systems (Anzai Respiratory Gating System; C‐RAD Sentinel) with respect to their applicability in combination with an Aquilion Large Bore CT scanner from Toshiba. The scanner used in our clinic does not make use of the full time dependent breathing signal, but only single trigger pulses indicating the beginning of a new breathing cycle. Hence the attached respiratory monitoring system is expected to deliver accurate online trigger pulse for each breathing cycle. The accuracy of the trigger pulses sent to the CT scanner has to be ensured by the selected respiratory monitoring system. Since a trigger pulse (output signal) of a respiratory monitoring system is a function of the measured breathing signal (input signal), the typical clinical range of the input signal is estimated for both examined respiratory monitoring systems. Both systems are analyzed based on the following parameters: time resolution, signal amplitude, noise, signal‐to‐noise ratio (SNR), signal linearity, trigger compatibility, and clinical examples. The Anzai system shows a better SNR (≥28 dB) than the Sentinel system (≥14.6 dB). In terms of compatibility with the cycle‐based image sorting algorithm of the Toshiba CT scanner, the Anzai system benefits from the possibility to generate cycle‐based triggers, whereas the Sentinel system is only able to generate amplitude‐based triggers. In clinical practice, the combination of a Toshiba CT scanner and the Anzai system will provide better results due to the compatibility of the image sorting and trigger release methods.

PACS numbers: 87.57.Q‐, 07.07.Df

## I. INTRODUCTION

In several radiotherapy treatment sites (e.g., lung, liver) respiration‐induced movements compromise the intention to deliver the prescribed dose to the tumor. The initial problem of tumor motion results in motion artifacts, which can be observed in the reconstructed images, erroneous Hounsfield unit (HU) values, and potentially insufficient dose coverage caused by incorrect motion estimation during delineation of the tumor volume. Hypofractionated stereotactic radiotherapy treatments are especially affected by these problems because of their particular small volumes.

To minimize these errors, different methods of CT acquisition were developed including respiration‐correlated computer tomography (RCCT)^1^, ^2^, ^3^ also referred to as 4D CT, respiratory‐gated CT acquisition,^4^, ^5^ or dynamic volume techniques.^6^, ^7^ Applicable methods depend to a certain degree on characteristics of the CT scanner. Dynamic volume techniques are limited to CT scanners with a large‐area 2D detector. Such detectors allow effective lengths of the scanned volume up to 160 mm.^(6)^ Thereby a whole volume scan can be acquired in about 0.35 sec. From multiple volume scans at different time points a dynamic volume can be reconstructed. During the acquisition of a respiratory‐gated CT, image data are collected only when the patient is in the previously defined respiratory state. The most prominent respiratory states for gated CT techniques are the end‐inspiration and the end‐expiration states. Another commonly used acquisition method is the 4D CT. A time resolved image dataset is reconstructed by oversampling — each image position is acquired multiple times during at least one complete breathing cycle — and retrospective sorting of all images into image stacks with the same respiratory state. The process of retrospective image sorting can be divided in two main groups using (a) amplitude‐based sorting algorithms or (b) phase‐based sorting algorithms. A more detailed classification is given by Guckenberger et al.^(8)^ The prerequisite common to all image sorting algorithms is that the images need to be tagged with their corresponding respiratory state. Therefore, respiratory monitoring systems are necessary to deliver information on the patient's respiratory state to the CT scanner. Inaccurate information on the respiratory state will result in errors during the retrospective image sorting and, consequently, in image artifacts. To avoid errors in image sorting it is important that the respiratory monitoring system delivers the exact information expected by CT scanner.

There are numerous respiratory monitoring systems on the market that uses different principles of measurement (e.g., Varian's RPM system; Philips Medical Systems' pneumatic bellows^(9)^). The aim of this study is to evaluate the applicability of the newly developed C‐RAD Sentinel system (C‐RAD AB, Uppsala, Sweden) in combination with the 4D CT mode of a Toshiba CT scanner (Toshiba Medical System Group, Tokyo, Japan). The second respiratory system available in our department — Anzai Respiratory Gating System (Anzai Medical Co. Ltd, Tokyo, Japan) — serves as a reference.

## II. MATERIALS AND METHODS

### A. Toshiba CT scanner

The scanner used in this study is a Toshiba Aquilion 16 Large Bore scanner including the AquilionLB Software Ver3.38ER005.^10^, ^11^ The 4D CT mode of the Toshiba scanner is derived from an electrocardiography mode (ECG), originally designed for cardiac imaging. With respect to the classification given by Guckenberger et al.,^(8)^ the implemented image sorting method is a special form of phase‐based sorting called cycle‐based sorting.

**Figure 1:**
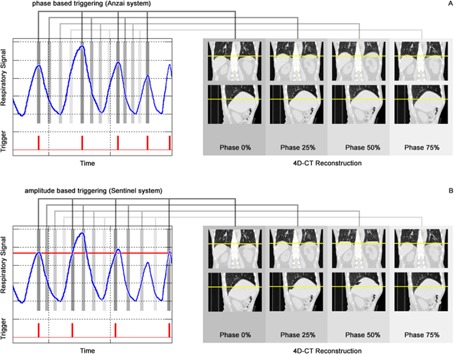
Scheme of the cycle‐based 4D CT mode as it is implemented in the Toshiba scanner. An exemplary respiratory signal with corresponding triggers delivered by (a) the Anzai System and (b) the Sentinel system is used to reconstruct n=4 respiratory phases. Triggers are expected to indicate the beginning of a new breathing cycle. Note that the amplitude‐based triggers delivered by the Sentinel system in (b) are set incorrect with respect to the length of a breathing cycle, including a complete inspiration and expiration. As a result, the image sorting in (b) is faulty.

For each breathing cycle including inspiration and expiration, the CT scanner expects a single trigger from the respiratory monitoring system indicating the beginning of the cycle. Images are then sorted into *n* equidistant phases between two recognized trigger pulses corresponding to *n* phases of the breathing cycle (Fig. 1(a)). The trigger pulses delivered to the CT scanner can be manipulated retrospectively, but in clinical use this is impractical and lacks accuracy, as the software does not show the time‐resolved breathing curve but solely the trigger pulses. Therefore, an important clinical requirement of each respiratory monitoring system is the ability to deliver cycle‐based online trigger pulses with high reliability.

### B. Anzai Respiratory Gating System

**Figure 2:**
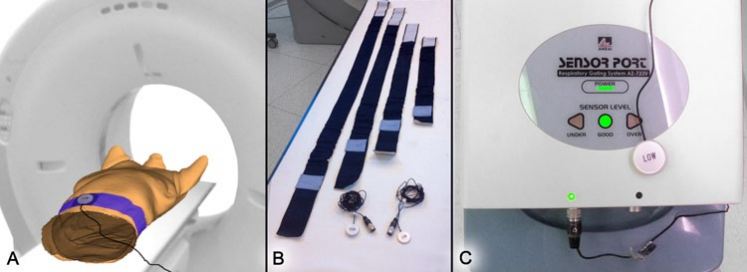
Application and components of the Anzai system: (a) pressure transducer placed at a patient's abdomen, attached by the fixation belt; (b) fixation belts in different sizes and both pressure transducers (low/high); (c) electrical amplifier with signal quality indicators for a low, good or high input signal.

The Anzai Respiratory Gating System (AZ‐733V Version 2.2H) consists of a fixation belt which is used to position a pressure transducer at the right upper quadrant of a patient's abdomen (Fig. 2(a)). Due to expansion and contraction of the belt during breathing, the pressure transducer delivers a digital voltage signal, which is amplified and then evaluated by the Anzai software. The system includes two pressure transducers (low/high) with different sensitivities for patients with shallow vs. deep respiration amplitudes, as well as four different sized fixation belts used to compensate different circumferences of different patients (Fig. 2(b)). Three LEDs indicate whether the pressure signal is optimal, too low, or too high, corresponding to a correct or incorrect application of the fixation belt (Fig. 2(c)). By selecting appropriate values for offset and gain of the electrical amplifier, the user is able to optimize the baseline and amplitude range of the breathing signal. Hence the monitored respiratory signal is a relative signal, which depends on the choice of the fixation belt and its application, as well as the used offset and gain values of the electrical amplifier. The software is able to trigger on different events such as inspiration‐peak, expiration‐peak or derived events, so that the system can be classified as a phase‐based triggering system (Fig. 1(a)). To detect these events correctly, a prediction model is implemented in the Anzai software.

### C. C‐RAD Sentinel laser scanner

**Figure 3:**
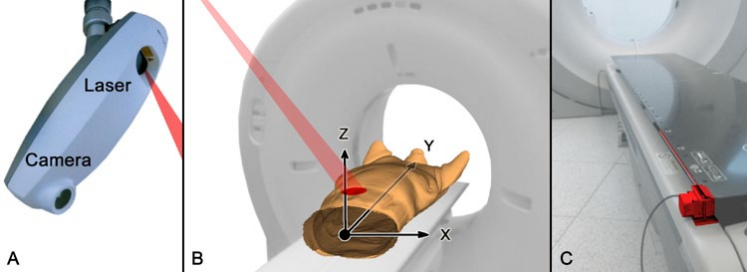
Installation and application of the Sentinel system: (a) Sentinel unit mounted at the ceiling; (b) patient setup and coordinate system; (c) the wire ruler (red) measures the couch position in Y direction and is mounted at the foot of the CT couch.

The Sentinel system consists of a laser which sweeps within a few seconds over the surface of the patient and a camera to detect the laser projections (Fig. 3(a)). From multiple projection lines, the software generates a static three‐dimensional model of the patient's surface. In addition to assistance in patient positioning, the Sentinel system contains a software module for 4D CT acquisition (c4D Version 4.5.0). By spotting a user‐defined region on the surface of a patient (Fig. 3(b)) and subsequent triangulation, an online surrogate signal of the patient's respiration can be acquired, which bases on the anterior/posterior movement (Z position) of the selected region. The resulting respiratory signal is scaled absolute and displayed in millimeters. By the use of two threshold values for the respiratory signal a window can be specified. Trigger events are released either on entrance or exit of the specified window or a combination of both. Since triggers are released at defined threshold values, the Sentinel system is an amplitude‐based triggering system (compare Fig. 1(b)). The c4D software does not provide any option to trigger on inspiration or expiration peaks.

There are at least two problems that have to be addressed to assure the accuracy of the recorded respiratory signal. First, the laser has to track the user‐specified measurement region correctly during couch movements. Therefore, the Sentinel system requires a wire ruler (Fig. 3(c)) that delivers the couch position to the laser positioning controller. Second, a CT couch typically consists of carbon fibers and is mounted with a slight pretension in positive Z direction to compensate for defections under the load of a patient. Depending on the patient's weight and the actual couch position, different defections in Z direction can be observed, which are superimposed on the respiratory signal. In order to correct this error, the Sentinel system makes use of a couch calibration profile. During the calibration process two profiles are recorded. The first one is used for daily check purposes only and, despite the Sentinel's QA Phantom, no additional weights are placed on the CT couch. The second couch calibration profile is recorded with the Sentinel's QA Check Phantom and additional calibration weights of 80 kg. The weights are placed along the couch to reproduce a typical patient load. This profile is used in the clinical mode for all patients regardless of their actual weight. The calibration weight of 70 kg recommended in the Sentinel's manual is increased by 10 kg to reproduce a representative patient weight in our clinic.

### D. Patient data

In this study two different patient datasets were analyzed. Set A contains 72 patient breathing curves and triggers recorded during clinical 4D CT sessions under free breathing using the Anzai system. The mean evaluated length of a respiratory signal was 46.8 sec, which corresponds to the mean time slot to acquire a 4D CT dataset using the Toshiba scanner. For all patients, a proper fixation belt was chosen and applied in such a way that the pressure transducer delivered an optimal signal corresponding to the LED indicators of the Anzai system. All breathing curves were recorded using the “low” sensor, which was applied at the right upper quadrant of a patient's abdomen. Set B contains breathing curves and triggers of 19 patients simultaneously recorded with the Anzai and the Sentinel system. This dataset was recorded during clinical 4D CT acquisition sessions using the Anzai system as trigger system for the CT scanner. The mean evaluated length of a respiratory signal was 43.1 sec. All application parameters for the Anzai system are the same as in Set A. Ideally the Sentinel's measurement region would have been placed at the same position as the Anzai pressure transducer. To avoid interference with the fixation belt, the individual measurement regions were placed in close proximity to the fixation belt.

### E. Analysis parameters

**Figure 4:**
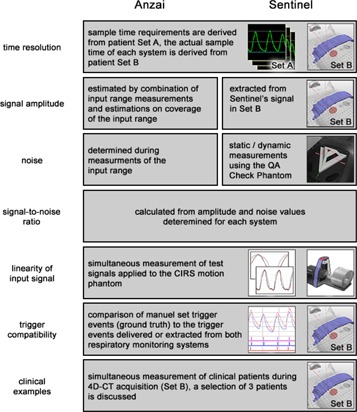
Overview of analysis parameters and methods to extract them for both respiratory monitoring systems.

To be able to compare both respiratory systems, comparable parameters must be defined and extracted for each system. Figure 4 gives an overview of the analysis parameters, as well as short information on the methods that are used to extract them.

#### E.1 Time resolution

Both respiratory monitoring systems must be able to sample respiratory signals without aliasing artifacts. Consequently, the sampling time should at least be half of the shortest respiration cycle, whereas a respiration cycle contains one complete inspiration end expiration phase (i.e., inspiration peak to inspiration peak). In practice, the sampling time should be less or equal than a tenth of the respiration cycle time. The latter is estimated in terms of the minimal respiration cycle times present in patient Set A, where the interpatient variability of minimal respiration cycle lengths ranged from 1.3 sec up to 8.7 sec. Consequently, the sampling time of both respiratory monitoring systems should match ideally 100 msec or less, as requirement. The actual sample times of the Anzai system and the Sentinel system are extracted from Set B.

#### E.2 Signal amplitude

The signal amplitude detected by the Anzai system is basically defined by the application of the fixation belt. The initial pressure signal is adapted by a tighter or looser application of the belt, and the mechanical pressure applied to the pressure transducer can only be estimated. In an ideal case the adapted pressure signal covers the whole input range of the pressure transducer. However, the only information about the input signal quality of the transducer is given by three indicator LEDs. To quantify the peak‐to‐peak amplitude of the Anzai system, the input range of each pressure transducer is determined by loading the transducer with increasing weights as long as the quality indicator is green. In a second step, it is assumed that the adaption done by the fixation belt is not ideal, but the signal applied to the transducer covers only a 1/3 of the input range. This assumption is somehow arbitrary because there is no way to determine the actual coverage of the input range beside the quality indicator LED's. For purposes of noise measurements, the input range was determined twice for each pressure transducer with different signal amplification values (gain) of 1 and 10.

Typical signal amplitudes for the Sentinel system are derived from patient Set B. For each patient, the respiratory signal is divided manually into inspiration and expiration phases. For each phase, the peak‐to‐peak amplitude between the inspiration peak and the expiration peak is calculated. The peak‐to‐peak signal amplitude for the Sentinel system is estimated by averaging the peak‐to‐peak amplitudes over all patients.

#### E.3 Noise

Both respiratory monitoring systems are exposed to different noise contributions. The noise amplitude of the Anzai system is determined during signal amplitude measurements. The measurement using the maximal signal amplification of 10 provides the maximal signal resolution and minimal input range. Hence, any noise contribution should be maximal and the noise amplitude is estimated from that measurement.

**Figure 5:**
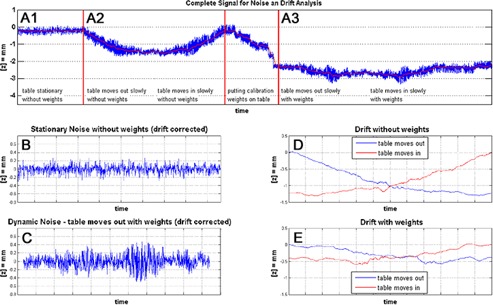
Noise and drift measurements of the Sentinel system: (top row) A1 stationary couch, no additional weights; A2 couch movement, no additional weights; A3 couch movement, additional calibration weights; between A2 and A3 the calibration weights are placed on the couch; (b) zoomed and offset corrected noise measurement of setting A1; (c) zoomed and drift corrected noise measurement of setting A3; (d) signal drift during couch movement without additional weights; and (e) with additional calibration weights corresponding to settings A2 and A3.

To examine different noise contributions on the Sentinel signal, the system is supposed to measure the surface (Z position) of the Sentinel's QA Check Phantom (see Fig. 5) in the clinical mode under the following conditions: A1) stationary couch without additional weights, A2) couch movement without additional weights, and A3) couch movement and additional weights. The QA Check Phantom is a rigid object with a mass of ~2 kg. The additional weights that are placed on the couch are exactly the same weights (80 kg) that are used to measure the couch calibration profile.

#### E.4 Signal‐to‐noise ratio

The signal‐to‐noise ratio for each system is calculated by using Eq. (1) and the root mean square (RMS) values of the estimated input and noise signals.

(1) $$SNR_{dB}=20\cdot {\text{log}}_{10}\left(\frac{RMS_{signal}}{RMS_{noise}}\right)$$

To facilitate calculations, it is assumed that both input signals are sinusoidal. Actually breathing signals are rarely sinusoidal. However, the assumption is applied to both respiratory systems and the results are comparable in that way. For sinusoidal signals with an amplitude, *a*, the RMS value can be formulated as:

(2) $$RMS_{signal}=a\cdot \frac{1}{\sqrt{2}}$$

Thereby the amplitude *a* is half the peak‐to‐peak amplitude.

The noise observed in the Anzai measurements is characterized by an alternating value in the least significant bit. Therefore, the RMS value is estimated by a pulse wave signals (Eq. (3)) with a pulse height *a* and the ratio *t/T* of pulse duration *t* and period time *T.* The ratio *t/T* is set to 0.5 since the error might occur with that probability.

(3) $$RMS_{noise}=a\cdot \sqrt{\frac{t}{T}}$$

Thereby the amplitude *a* equals the peak‐to‐peak amplitude.

For the calculation of the Sentinel's ${\text{RMS}}_{\text{noise}}$ value, the more general form (see Eq. (4)) is used in combination with the three settings A1), A2), and A3). Whereas *n* is the number of measured points and $x_{i}$ represents the noise value at point i in the measurement.

(4) $$RMS_{noise}=\sqrt[{2}]{\frac{1}{n}\sum_{i=1}^{n}x_{i}^{2}}$$

#### E.5 Signal linearity

Each of the respiratory monitoring system is expected to have a linear dependency between its input and output signal to avoid the introduction of additional distortion on the correlation of tumor motion and the monitored respiratory signal. To test both systems, two breathing patterns are applied to a dynamic thorax phantom (Model 008A; Computerized Imaging Reference Systems, Inc., Norfolk, VA) and measured simultaneously with the Anzai and the Sentinel system. Pattern A is a sinusoidal signal with a cycle length of 4 sec and peak‐to‐peak amplitude of 1 cm. Pattern B is a breathing curve from patient Set A rescaled to a maximal excursion of 1 cm between the global maximum and minimum of the pattern. Both patterns are chosen to reproduce a patient‐like breathing signal for both respiratory monitoring systems.

#### E.6 Trigger compatibility

A major requirement to all respiratory monitoring systems used in combination with the Toshiba CT scanner is a high reliability of the online delivered cycle‐based trigger events. Thereby the compatibility of trigger events includes the requirement that all triggers are released at the same respiratory phase, as well as the correct number of released triggers.

The Anzai system is able to deliver triggers at the same respiratory phase, by triggering on the inspiration peak. The Sentinel system delivers amplitude‐based triggers only. For periodic and piecewise monotonic signals like a sinusoidal signal, the signal's phase is correlated with the signal's absolute value. In such a case, the amplitude‐based triggers delivered by the Sentinel system are correlated to the same respiratory phase. However, respiratory signals are not strictly periodic, but quasi‐periodic, signals that vary in amplitude as well as in cycle length. Therefore, the triggers delivered by the Sentinel system are not necessarily correlated to the same respiration phase (Fig. 1(b)) as it is expected by the Toshiba CT scanner and phase shifts are introduced. As the user is interested in the most extreme tumor positions during the respiration cycle, the amplitude threshold of the Sentinel system should be set as close as possible to the value of the inspiration peak to minimize phase shift errors for the maximum inspiration phase.

To test both respiratory systems with respect to the correct number of released triggers for each respiratory curve in patient Set B, a ground truth trigger signal is created by setting trigger events manually at the beginning of each respiratory cycle (inspiration peak). The Anzai system is set up to trigger on the inspiration peak, too. Since the trigger events from the Sentinel system cannot be saved in a data file, the triggers are extracted from the respiratory curve using a threshold value. Therefore, all breathing curves of the Sentinel system are imported into MATLAB (MathWorks, Natick, MA) and rescaled to an arbitrary scale. To account for instabilities in signal amplitudes, two different threshold values are used (80% and 60% of the signal maximum) to extract triggers as they would be delivered by the Sentinel system. The absolute number of trigger events delivered by each respiratory system is compared to the number of ground truth trigger events expected for each patient.

#### E.7 Clinical examples

In order to discuss the performance of both respiratory systems on clinical data, three exemplary patients from the patient Set B are selected. The breathing signal of each respiratory monitoring system, as well as the patient setup, is visualized and the examples are discussed.

## III. RESULTS

### A. Time resolution

The sample time for both respiratory systems should match ≤100 msec. Both trigger systems meet that requirement. The Anzai system is the fastest system with a constant sample acquisition every 25 msec. The sample times of the Sentinel system are not constant. A mean sample time of 42.2±0.9 msec can be observed in the data.

### B. Signal amplitude

The measurements on the input ranges and estimations about the signal amplitudes of the Anzai system are summarized in Table 1. For instance, the peak‐to‐peak amplitude for the pressure transducer “low” and a gain of 10 results in 9.3 g (input range of 28 g multiplied by the estimated input range coverage of 1/3). The corresponding ${\text{RMS}}_{\text{signal}}$ values are calculated using Eq. (2).

The signal amplitude for the Sentinel system is calculated from the respiratory signals in patient Set B. The mean peak‐to‐peak signal amplitude over all patients in Set B is 7.7±4.8 mm (Table 2). Two patients (#6, #16) are excluded from the calculation due to extremely small signal amplitudes. For these patients, the region observed by the Sentinel system was placed next to the Anzai belt but not at the abdomen, but rather at the patient's chest. The corresponding ${\text{RMS}}_{\text{signal}}$ value is calculated from the mean signal amplitude by the use of Eq. (2). The resulting value for the Sentinel system is ${\text{RMS}}_{\text{signal}}=2.7\;\text{mm}$.

**Table 1:** Overview of the Anzai pressure transducers and the calculation base of the corresponding SNR values

*Pressure Transducer*	*Signal Amplification (gain)*	Input Range/Resolution (measured) [mass]=g	Peak‐To‐Peak Signal Amplitude (coverage 1/3) [mass]=g	${\text{RMS}}_{\text{signal}}\;[\text{mass}]=g$	${\text{RMS}}_{\text{noise}}\;[\text{mass}]=g$
Low	1.0	170/1.13	56.7	20.0	0.80
	10.0	28/0.18	9.3	3.3	0.13
High	1.0	280/1.85	93.3	33.0	1.31
	10.0	50/0.33	16.7	5.9	0.23

**Table 2:** Evaluation results of Patient Set B with respect to the Sentinel's input signal amplitude (surface motion) and the absolute number of expected triggers (ground truth) and the delivered triggers for each setup

*Patient from Set B*	Peak‐To‐Peak Signal Amplitude [Avg.±Std]=mm	*Ground Truth Triggers*	*Anzai Triggers*	*Sentinel Triggers* (thr=80%)	*Sentinel Triggers* (thr=60%)
1	4.2±0.7	9	9	6	10
2	7.0±1.9	13	9	3	9
3	3.7±0.4	14	14	5	15
4	11.5±2.7	12	10	7	13
5	4.9±1.0	11	11	5	8
6^a^	0.8±0.2	15	15	27	–
7	6.6±0.7	14	14	5	14
8	7.1±0.5	11	10	11	11
9	6.4±0.7	10	9	8	10
10	26.4±2.0	8	8	8	8
11	5.3±0.5	16	14	12	16
12	13.4±2.0	12	11	6	12
13	11.8±2.2	10	10	4	9
14	5.0±0.5	13	13	13	13
15	5.0±0.7	10	10	11	15
16^a^	2.3±0.2	13	13	29	–
17	7.6±2.5	15	11	4	11
18	6.9±1.0	8	8	3	8
19	5.7±0.3	9	9	9	10
Mean	7.7±4.8				

^a^ Patient is removed from the calculation of the overall mean peak‐to‐peak signal amplitude due to an improper placement of the region observed by the Sentinel system.

Due to different principles in measurement and different units of the signals, a direct comparison of the signal amplitudes or the RMS values is not possible.

### C. Noise

The noise observed in the Anzai measurements is characterized by an alternating value in the least significant bit. The resulting amplitude, *a*, of that alternating least significant bit equals the resolution of the Anzai system. Values for the resolution are given in Table 1 and range from 0.18 g up to 1.85 g. By the use of Eq. (3) the corresponding ${\text{RMS}}_{\text{noise}}$ values are calculated, which are also given in Table 1.

To examine different error contributions on the Sentinel signal, noise was measured for three different settings. Figure 5 shows the complete measurement, where the settings (top row of figure) A1, A2, and A3 correspond to the sections of the same name. Figure 5(b) shows a zoomed and offset corrected image of setting A1. Depending on the load of the scanner couch, different signal drifts can be observed in Fig. 5(d) (weightless) and 5(e) (with calibration weights).

The drift signals are the smoothed signals of the settings A2 and A3. Beside the drift, there is an increase in noise when the couch is moved, compared to the stationary noise (Fig. 5(b) and 5(c))). The measurement is exposed to noise from the electronic of the wire ruler and the laser projection. On the other hand, external errors are introduced by the defection of the CT couch and its movement. A couch defection depending on the actual load affects the measurement and appears as an additional signal drift during couch movement. Typical noise amplitudes results in calculation errors for the Z position of 0.1 millimeter, whereas drift errors are an order of magnitude greater than the noise. The overall ${\text{RMS}}_{\text{noise}}$, including noise and drift, results in ${\text{RMS}}_{\text{noise}}=1.07\;\text{mm}$ for weightless and ${\text{RMS}}_{\text{noise}}=0.41\;\text{mm}$ for the loaded couch. Noise and drift errors depend on the actual deviation of the patient's weight from the calibration weight (80 kg). A conservative estimation on a deviation of ~11 kg (actual patient weight between 69 kg and 91 kg) results in an estimation for the Sentinel's noise by ${\text{RMS}}_{\text{noise}}=0.5\;\text{mm}$.

### D. Signal‐to‐noise ratio

The SNR values for both respiratory monitoring systems are calculated by the use of Eq. (1). The Anzai system shows an SNR value of ~28 dB for all measured combinations of pressure transducers and selected signal amplifications. According to the figures from Table 1, the SNR values for the pressure transducer “low” result in 28.0 dB using a signal amplification of 1 and 28.3 dB for a signal amplification of 10. By analogy, the SNR value for the pressure transducer “high” result in 28.0 dB for both selected signal amplifications.

The SNR value for the Sentinel system is 14.6 dB.

### E. Signal linearity

**Figure 6:**
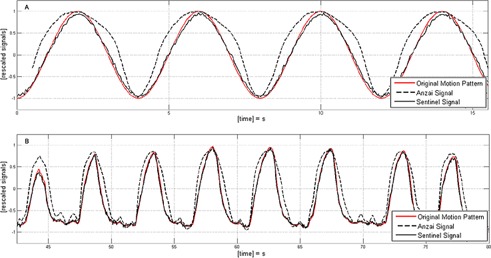
Different motion patterns applied to the motion phantom and resulting signals detected by both respiratory monitoring systems. Patterns: (a) sinusoidal motion with a cycle length $t_{\text{cycle}}=4\;\text{sec}$ and a peak‐to‐peak amplitude $s_{\text{pp}}=1\;\text{cm}$; (b) breathing pattern of a patient rescaled to an amplitude $s_{\text{max}‐\text{min}}=1\;\text{cm}$.

**Figure 7:**
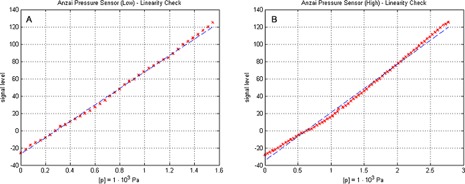
Sensor response on applied pressures for (a) the pressure transducer “low” and (b) the pressure transducer “high”. The linear regression fit is shown as blue line for both transducers.

The comparison of two test signals applied to both respiratory monitoring systems shows a nonlinearity (Fig. 6) introduced by the Anzai system. Although the fixation belt was double‐checked and the quality indicator LEDs reported a perfect signal adaption, it seemed that the pressure sensor reached saturation or that the fixation belt introduced additional forces to the pressure sensor. Further analysis showed that both sensors (low/high) generate a nearly linear response (Fig. 7). Hence the introduced nonlinearity is generated by the rubber‐like elasticity of the fixation belt. The magnitude of the nonlinearity depends on the size of the belt and the applied forces. With decreasing belt size and increasing signal amplitude the nonlinear effects increases. In the signal linearity measurements, the smallest belt size was chosen to mount the pressure transducer on the motion phantom. Therefore, the observed magnitude of nonlinearity is higher than in clinical data.

### F. Trigger compatibility

**Figure 8:**
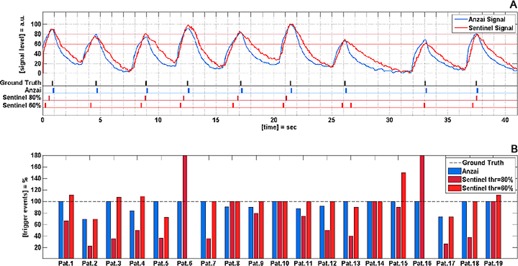
Evaluation of trigger reliability for both respiratory monitoring systems: (a) respiratory signals for both systems and the corresponding trigger events for a Patient #1; (b) comparison of delivered trigger events and ground truth triggers for all patients in Patient Set B.

Both systems show errors in the trigger release (Fig 8(a)). However, the Anzai system delivers the more accurate trigger signal with respect to the cycle‐based triggers expected by the Toshiba CT. In 12 of 19 patient curves, the Anzai system releases a correct number of triggers by the automatic detection of the inspiration peak. The fact that the triggers are released at the inspiration peak assures that the length of a breathing cycle can be calculated from the Toshiba CT correctly.

The absolute number of trigger events delivered by the Sentinel system is less accurate. For a threshold level of 80%, only 4 of 19 patients show a correct number of trigger events. By a decreased threshold level of 60%, the number of patients for whom a correct number of triggers is released increases to 8 of 19 patients. In addition to missed breathing cycles due to amplitudes lower than the threshold value, noise on the Sentinel's signal leads to trigger events that are not correlated with a breathing cycle. That is why for some patients more triggers are released than expected from the ground truth trigger signal (compare Table 2 and Fig. 8(b)).

### G. Clinical examples

**Figure 9:**
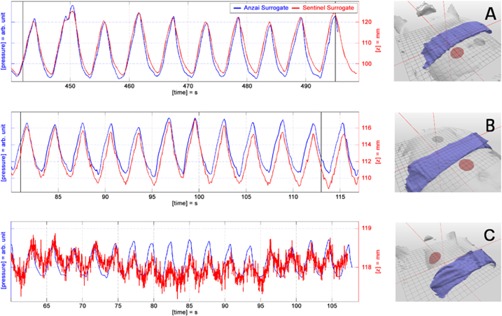
Three exemplary patients with respiratory signals recorded simultaneously with the Anzai and the Sentinel system. The right‐hand pictures show the patient setup with the Anzai belt (blue) and the Sentinel region (red). Patterns: (a) regular breathing patient with large signal amplitudes; (b) drift error of the Sentinel system resulting from inappropriate correction of couch defection; (c) small surrogate signal due to shallow breathing and an inappropriate selection of the Sentinel's measurement area.

To visualize the effects on the clinical data, three exemplary patients were selected from Set B. For each of them, the measured Anzai signal, as well as the Sentinel signal, is plotted in one diagram followed by a small picture of the corresponding patient setup (Fig. 9).

For Patient A, a well‐adapted signal can be observed for both respiratory monitoring systems, caused by a regular respiration with good intrafractional amplitude stability and large signal amplitudes. The signal from the Anzai system shows steeper signal edges and overshooting of the signal at inspiration peaks. Supposedly, both effects result mainly from the nonlinearity introduced by the fixation belt. Although the respiration curve is close to an ideal signal, the generation of triggers by the use of an amplitude threshold (Sentinel) will introduce phase shifts into reconstruction data. Due to amplitude variability, the selected threshold value does not correlate with the same respiration phase.

Patient B shows a characteristic drift artifact of the Sentinel system, caused by the use of inappropriate values for the correction of couch defection. The absolute peak‐to‐peak amplitudes of the Sentinel system range between 5 to 7 mm and show good agreement to the figures presented in Table 2. Although the signal drift is only about 1 mm, amplitude based‐triggers would introduce a phase shift and thereby reconstruction artifacts in the 4D CT. Cycle‐based triggers relying on the detection of the inspiration peak (Anzai) would provide better results in reconstruction.

The effect of different SNR values for both respiratory monitoring systems is demonstrated by Patient C, who exhibits an extreme shallow respiration. The absolute signal from the Sentinel system ranges only between 0.5 to 1 mm. The two error contributions — noise and signal drift — are added onto the respiration signal and make it impossible to estimate the respiratory cycle based on an amplitude threshold as it is used by the Sentinel system. Besides, it is obvious that the selected region to extract the surrogate signal for the Sentinel system is not a good choice in this example. The recommended region to extract surrogate signals is the right upper quadrant of the human abdomen.

## IV. DISCUSSION

Toshiba's 4D CT mode makes use of a cycle‐based image sorting algorithm and therefore expects cycle‐based trigger pulses. Erroneous triggers have to be corrected retrospectively on the Toshiba console, otherwise they will lead to reconstruction artifacts. A retrospective correction of the trigger signal on the Toshiba console is impractical, since the software does not show a respiratory curve, but solely the received triggers. That is why any respiratory monitoring system used in combination with the Toshiba CT is required to deliver online, cycle‐based triggers with high reliability.

The Anzai system is able to generate correct cycle‐based trigger events for a majority of patients (12/19). The Sentinel system supports amplitude‐based triggering only. That causes two problems. First, the signal amplitude is sensitive to noise and drift errors, which directly implies that trigger events are sensitive to such errors. Due to the noise on the respiratory signal of the Sentinel system, the number of released triggers may exceed the number of triggers expected from the respiratory signal (e.g., Fig 8(b)). Second, the choice of a proper threshold value is a critical decision. As shown in Fig. 8(b), the threshold value will influence the number of released triggers. In cases where the inspiration peak is below the threshold value, no trigger will be released. A decrease of the threshold value from 80% to 60% will increase the number of patients with a correct detected number of triggers from 4 to 8 out of 19 patients (compare Fig. 8(b)). However, by decreasing the threshold value from 80% to 60%, the uncertainty about the trigger assigned respiratory phase will grow. This effect can be observed in Fig. 8(a) at time points between 20 sec and 27 sec. By using a threshold value of 80% the inspiration peak at 26 sec is not detected. The lower threshold of 60% assures the release of two trigger events; nevertheless, the first trigger is released in a midinspiration phase and the second trigger is released just under the inspiration peak. Therefore, the two triggers do not represent a breathing cycle as it is expected from the Toshiba CT scanner. In this study, the trigger events delivered by the Sentinel system are less accurate with respect to the absolute number of delivered triggers and the possibility to extract the correct length of a breathing cycle from the delivered triggers. Besides, the selection of the proper threshold value has to be done before the acquisition of the 4D CT, since the Toshiba CT scanner expects online triggers. Therefore, it is unknown what the respiratory signal will exactly look like during the 4D CT acquisition and whether the selected threshold value is a good choice.

To fulfill the requirement of reliable, cycle‐base delivered online triggers, the Sentinel system requires a high stability in respiratory amplitudes, since only then amplitude‐based triggers are comparable to cycle‐based triggers. Amplitude stability can be improved by visual feedback of the respiratory signal to the patient (see Kini et al.^(12)^). One option to establish a visual feedback would be the application of the video googles available for the Sentinel system.

Regardless of the chosen respiratory monitoring system, every deviation in respiration amplitude will cause motion artifacts in 4D CT reconstructions, since the reconstructed 4D CT contains multiple respiration cycles that are combined to one single respiration cycle. Therefore, amplitude stability is a general requirement for 4D CT acquisition. However, amplitude stability is also an essential requirement of the Sentinel system to be able to deliver triggers that are comparable to cycle‐based triggers, whereas the Anzai system does not necessarily require highly stable amplitudes of the respiratory signal to release correct cycle‐based trigger events.

Referring to requirements given by the input signal of each respiratory monitoring system, a time resolution of 100 msec is recommended, which is met by both respiratory monitoring systems.

The SNR value for the Anzai system is clearly better (≥28 dB) than the Sentinel's SNR (≥14.6 dB). In order to improve the correct number of delivered triggers delivered by the Sentinel system, it is desirable that the Sentinel's SNR is improved by the manufacturer. This can be achieved, for example, by the use of low pass filters and by fine‐tuning the correction for the weight and position dependent couch defection, which is the major error contribution. The software should be adapted such that multiple couch calibration profiles are acquired and used depending on the actual weight of a patient. Another way to improve the compatibility between the Sentinel system and the Toshiba CT scanner would be the implementation of a cycle‐based online triggering option (inspiration peak detection) in the c4D software.

The Anzai system introduces a nonlinear dependency between the actual motion and the measured signal, mainly caused by rubber elasticity of the fixation belt. The magnitude of this additional error is hardly predictable and depends on the fixation belt and the applied forces. For triggering on the inspiration peak, the introduced nonlinearity does not matter. However, in gating applications, that nonlinearity of the respiratory signal compromises the gating window. That is why the Sentinel system is the better choice for gating applications in radiotherapy, beside the fact that the Sentinel system uses an absolute respiratory signal in comparison to the relative signal of the Anzai system.

Another advantage of the Sentinel system is the contactless measurement of the respiratory signal. In contrast to that, the fixation belt of the Anzai system may constrain the free breathing of a patient and thereby introduce an unintentional interference between the measuring device and the patient's breathing. This is a potential error in cases where the fixation belt is used during 4D CT acquisition but not during treatment, because estimations on tumor movement might be incorrect.

## V. CONCLUSIONS

In clinical practice the combination of a Toshiba CT scanner and the Anzai system will provide better results in 4D CT reconstruction due to the compatibility of methods in image sorting (CT) and trigger release (respiratory monitoring system). To improve the performance of the Sentinel system in combination with a Toshiba CT scanner, a cycle‐based trigger option should be added to the c4D software by the vendor. In the 4D CT acquisition using the Toshiba CT scanner and the current version of the Sentinel system, a visual feedback of the respiratory signal is strongly recommended. By visual feedback of the respiratory signal to the patient, it is possible to improve amplitude stability, which is a prerequisite to deliver triggers comparable to cycle‐based triggers using the amplitude triggering method of the Sentinel system. Another benefit of the amplitude stability accomplished by visual feedback would be to address the intrinsic problems caused by irregular breathing in the cycle‐based 4D CT reconstructions.

## Supporting information

Supplementary Material
