# Supplementary figures and images for: Technical evaluation of different respiratory monitoring systems used for 4D CT acquisition under free breathing
Source: J Appl Clin Med Phys. 2015 Mar 8;16(2):334–49. doi: 10.1120/jacmp.v16i2.4917 (PMC5690076; doi:10.1120/jacmp.v16i2.4917)

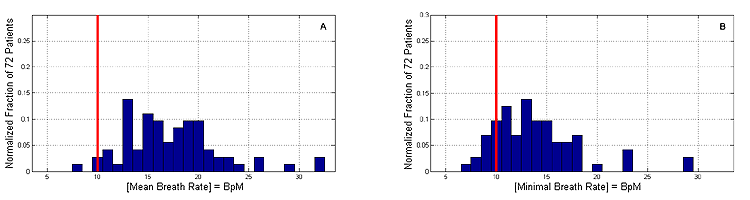

Supplement: Supplementary file 1 — Supplementary Material [file ACM2-16-334-s001.png]
